# A review of HTA guidelines on societal and novel value elements

**DOI:** 10.1017/S026646232300017X

**Published:** 2023-05-25

**Authors:** Rachel Milstein Breslau, Joshua T. Cohen, Jose Diaz, Bill Malcolm, Peter J. Neumann

**Affiliations:** 1 Center for the Evaluation of Value and Risk in Health, Institute for Clinical Research and Health Policy Studies, Tufts Medical Center, Boston, MA, USA; 2 Bristol-Myers Squibb Pharmaceuticals Ltd., Uxbridge, UK

**Keywords:** Health technology assessment, cost-effectiveness analysis, novelvalue elements, societal perspective

## Abstract

**Objectives:**

Health technology assessment (HTA) organizations vary in terms of how they conduct assessments. We assess whether and to what extent HTA bodies have adopted societal and novel elements of value in their economic evaluations.

**Methods:**

After categorizing “societal” and “novel” elements of value, we reviewed fifty-three HTA guidelines. We collected data on whether each guideline mentioned each societal or novel element of value, and if so, whether the guideline recommended the element’s inclusion in the base case, sensitivity analysis, or qualitative discussion in the HTA.

**Results:**

The HTA guidelines mention on average 5.9 of the twenty-one societal and novel value elements we identified (range 0–16), including 2.3 of the ten societal elements and 3.3 of the eleven novel value elements. Only four value elements (productivity, family spillover, equity, and transportation) appear in over half of the HTA guidelines, whereas thirteen value elements are mentioned in fewer than one-sixth of the guidelines, and two elements receive no mention. Most guidelines do not recommend value element inclusion in the base case, sensitivity analysis, or qualitative discussion in the HTA.

**Conclusions:**

Ideally, more HTA organizations will adopt guidelines for measuring societal and novel value elements, including analytic considerations. Importantly, simply recommending in guidelines that HTA bodies consider novel elements may not lead to their incorporation into assessments or ultimate decision making.

## Introduction

It is well known that health technology assessment (HTA) organizations vary in terms of how they conduct assessments (1). This paper focuses on whether and how HTA bodies have adopted “societal” and so-called *novel* elements of value in their economic evaluations. Following recent guidance, we define HTA as “a multidisciplinary process that uses explicit methods to determine the value of a health technology at different points in its lifecycle…” and whose purpose is “to inform decision making in order to promote an equitable, efficient, high-quality health system” (2).

By *societal* elements, we mean components beyond health impacts to the treated individual and costs beyond those incurred by the healthcare sector to deliver those interventions. By *novel* elements, we denote certain additional elements (e.g., insurance value, severity modifiers, and value of hope), that may reflect value but are not normally captured in conventional cost-effectiveness analyses (CEAs) and were highlighted by the ISPOR Special Task Force on U.S. value assessments (3).

We acknowledge at the outset some arbitrariness in the way we categorized “societal” versus “novel” elements or included them at all and that other investigators might categorize them differently. HTA organizations and consensus panels have differed in the manner in which they have defined and included societal elements, and some bodies that have mainly used a payer perspective have sometimes included certain societal elements (4). Moreover, research on defining value elements continues to evolve, particularly in light of the COVID-19 pandemic (5). Nonetheless, given the growing importance of HTA organizations in informing drug pricing and reimbursement, and ongoing debates about appropriate methods for value assessment, it is important to explore whether and how HTAs are formally incorporating these elements into evaluations. Our aim was to investigate whether and to what extent HTA bodies have adopted “societal” and *novel* elements of value in their economic evaluations, and whether such adoption has increased over time. We hypothesized that HTA guidelines published more recently would include a greater proportion of the value elements because of the heightened discussion of and research on these elements in recent years.

## Methods

### Identification of societal and novel value elements

*Societal elements.* Health economists have long recognized the importance of “perspective” in economic evaluations and have broadly distinguished a healthcare sector (or payer) perspective from a societal perspective. As noted, for example, by the Second Panel on Cost-Effectiveness in Health and Medicine, the healthcare sector perspective “reflects the view of a decision maker whose responsibility rests only within that sector”; in contrast, a “societal perspective reflects the perspective of a decision maker whose intention is to make decisions about the broad allocation of resources across the entire population” (4).

The perspective taken in an analysis determines the components to consider. A healthcare payer perspective typically includes the medical costs borne by public or private payers and a healthcare sector perspective includes these costs in addition to out-of-pocket health costs shouldered by patients. (Some analysts focus on a healthcare payer perspective, including only those costs directly affecting the payer.) While definitions of societal elements differ somewhat across jurisdictions and guidelines, they often include elements, such as time costs incurred by patients in seeking and receiving care, time costs incurred by informal (unpaid) caregivers, transportation costs, effects on future productivity and consumption, and other costs and effects outside the healthcare sector (4). The Second Panel recommended that CEAs include an impact inventory, which enumerates societal elements in the informal healthcare sector (patient-time costs, unpaid caregiver-time costs, and transportation costs) and non-healthcare sectors (productivity, consumption, social services, legal or criminal justice, education, housing, and environment) (4).

Motivated by the impacts of COVID-19, we also included *economic activity* and *healthcare capacity* as elements of value in this study. By *economic activity*, we mean the impact a disease can have on supply and demand in the broader economy and the subsequent changes a treatment for that disease might induce. By *healthcare capacity*, we mean the effects of the strain on the healthcare system when a disease causes it to approach or reach its capacity to treat patients.

*Novel elements.* The term “novel” elements stems from the 2018 report of the ISPOR Strategic Task Force (STF), which called attention to other elements typically not included in conventional CEAs (3). The STF identified two common, but inconsistently included value elements (labor productivity and adherence-improving factors), which we will categorize as novel elements in this study, and eight newer elements (reduction of uncertainty, fear of contagion, insurance value, severity of disease, value of hope, real option value, equity, and scientific spillovers) (3). Some of the novel elements reflect the idea that quality-adjusted life-years (QALYs) may not account for people’s preferences to avoid risk and uncertainty (6). For example, even if it does not improve outcomes on average, individuals may prefer a drug with more predictable benefits; QALYs would not account for this preference. Furthermore, QALYs do not distinguish between a long period spent in a moderately diminished health state and a shorter period spent in a more severe health state. In reality, people may prioritize severe disease treatments, whether or not that is consistent with QALY maximization. In addition, individuals may place a premium on therapies that offer a small chance of substantial health gains, a phenomenon sometimes called the “value of hope” (7–9). That is, many patients would be willing to gamble on a risky but promising cancer drug, even if a QALY-maximizing strategy would not recognize such preferences (7). Building on our previous work on lifecycle drug pricing, we also assessed HTA guideline inclusion of drug *genericization*, an allowance for future generic drug entry and subsequent price declines (10). Though it is not a value element, *per se*, but rather a methodological consideration for calculating future costs, we include drug genericization here for completeness because it is an often omitted and debated component of value assessment (Table 1).

**Table 1.** List of societal and novel value elements considered

Societal value elements	Novel value elements
Consumption	Adherence-improving factors
Economic activity	Equity
Education	Fear of contagion
Environment	Genericization
Family spillover	Insurance value
Healthcare system capacity	Productivity
Housing	Real option value
Legal	Reduction of uncertainty
Social services	Scientific spillover
Transportation	Severity of disease
	Value of hope

### Selection of HTA guidelines

We reviewed fifty-three HTA guidelines to determine whether and how they referenced societal and novel elements of value (4;11–62). The sample draws upon our prior work reviewing HTA guidelines on other aspects of CEA (63), and is based on an updated search of the literature (as of December 2021) as well as a review of web sites of HTA organizations and the ISPOR inventory of “Pharmacoeconomic Guidelines Around the World” (64). As in our prior research, for completeness, we included seven notable nongovernment, United States, and international HTA guidelines (from the Institute for Clinical and Economic Review (ICER), the ISPOR Drug Cost Task Force, the Second Panel, the Academy of Managed Care Pharmacy, WellPoint, Drummond et al., and Wilkinson et al.) (4;11;52;54;59–61). While these practices or guidelines are not produced by government entities, they are well-known and commonly cited references for CEAs in the United States and internationally.

The fifty-three guidelines in our final sample represent fifty-two countries. In three cases (the MERCOSUR nations of Argentina, Brazil, Paraguay, and Uruguay (65); the Baltic states of Latvia, Estonia, and Lithuania (14); and the United Kingdom nations of England and Wales (24)), multiple countries share a single guideline. In other cases, the guidelines do not represent a specific country (ISPOR Drug Cost Task Force, the Second Panel, The Academy of Managed Care Pharmacy, WellPoint, Drummond et al., and Wilkinson et al.) (4;11;52;54;59–61) or multiple guidelines represent the same country (MERCOSUR guideline (65) and Brazilian guideline (62)) (Table 2 and Appendix Table 1 in the Supplementary Material).

**Table 2.** Characteristics of HTA guidelines by country income

World bank income category	Number of HTA guidelines	Number (%) of HTA guidelines mentioning any value element	Countries with HTA guidelines mentioning any value element
High income	34	23 (67%)	Australia, Belgium, Canada, Chile, Croatia, Czech Republic, Denmark, England, France, Germany, Hungary, ICER, Ireland, New Zealand, Norway, Poland, Scotland, Slovenia, South Korea, Spain, Switzerland, Taiwan
Upper-middle income	10	8 (80%)	Brazil, China, Colombia, Cuba, Malaysia, MERCOSUR, Mexico, Thailand
Lower-middle income	4	3 (75%)	Egypt, Indonesia, Philippines
Low income	0	NA	NA
Non-country	6	5 (83%)	AMCP, Drummond et al., ISPOR, the Second Panel, Wilkinson et al.
Total	53	38 (72%)	

Abbreviations: AMCP, Academy of Managed Care Pharmacy; HTA, health technology assessment; ICER, Institute for Clinical and Economic Review; ISPOR, The Professional Society for Health Economics and Outcomes Research; MERCOSUR, Common Markets for South Latin America (Argentina, Brazil Paraguay, and Uruguay); NA, not applicable.

### Development of data collection form

We created a data collection form to record salient information. We collected data on whether each guideline mentioned each societal or novel element of value, and if so, whether the guideline recommended the element’s inclusion in the base case, sensitivity analysis, or qualitative discussion in the HTA. These categories are not mutually exclusive or exhaustive. A guideline could recommend an element’s inclusion in multiple categories or mention an element and not recommend its inclusion in any of these categories (Appendix Table 2 in the Supplementary Material).

### Data collection

Two researchers pilot-tested the form on five randomly-selected HTA body guidelines and made minor changes to the form based on the results. Appendix Table 2 in the Supplementary Material includes the final form. One researcher then abstracted data on the remaining HTA body guidelines, which together with the five pilot guidelines we retained, yielded a final sample of fifty-three guidelines. The researcher abstracted data independently and met with other researchers to resolve any uncertainty.

## Results

### Characteristics of HTA guidelines

The HTA organizations in our sample included forty-six government agencies (12–51;53;55–59;62), six independent organizations (4;11;52;54;60;61), and one U.S. private payer (Wellpoint) (59). The government organizations come from countries with a range of per capita incomes, including high-income countries (thirty-four guidelines) (11–17;20–22;24–28;30–33;35;37–45;47–50;52;54;57;59–62), upper middle-income (ten guidelines) (18;19;34;36;46;51;55;56;62;65), and lower-middle (four guidelines) (23;29;53;58) (Table 2). Government and foundation funding supports most of the work conducted by these HTA organizations. The HTA organizations published the guidelines included in our sample from 2002 to 2022. The guidelines make recommendations pertaining to different types of clinical and economic evaluations, including cost–utility analysis, CEA, cost–benefit analysis, cost–consequence analysis, cost minimization analysis, and budget impact analysis. Most of the guidelines are from high-income countries and most mentioned at least one of the value elements we considered (Table 2). Forty guidelines recommend a societal perspective, thirty-four recommend a healthcare payer perspective, and nine recommend a healthcare sector perspective (not mutually exclusive).

### HTA guidelines’ mention of societal and novel elements

HTA guidelines vary in terms of the number and type of value elements they mention (Appendix Table 3 in the Supplementary Material). Most HTA guidelines mention few societal and novel value elements. The HTA guidelines in our sample mentioned on average 5.9 of the twenty-one value elements we identified, ranging from 0 to 16. The HTA guidelines mentioned on average 2.3 of the ten societal elements we identified and 3.3 of the eleven novel value elements. The frequency with which the value elements appear in the HTA guidelines also varies widely (Table 3). Only four value elements (productivity, family spillover, equity, and transportation) appear in over half of the HTA guidelines, thirteen value elements are mentioned in fewer than one-sixth of the guidelines, and two elements receive no mention (Table 3).

**Table 3.** HTA organizations inclusion of value elements

Value element	HTA guideline mentioned value element^a^	HTA guideline recommended value element inclusion in^a^:		
		Base case analysis	Sensitivity analysis	Qualitative discussion
Productivity	42 (79%)	14 (26%)	23 (43%)	2 (4%)
Family spillover	41 (77%)	18 (34%)	18 (34%)	5 (9%)
Equity	35 (66%)	0 (0%)	5 (9%)	23 (43%)
Transportation	27 (51%)	14 (26%)	10 (19%)	0 (0%)
Adherence-improving factors	25 (47%)	5 (9%)	3 (6%)	5 (9%)
Severity of disease	21 (40%)	0 (0%)	3 (6%)	4 (8%)
Social services	15 (28%)	7 (13%)	4 (8%)	0 (0%)
Genericization	15 (28%)	9 (17%)	7 (13%)	1 (2%)
Education	8 (15%)	2 (4%)	3 (6%)	0 (0%)
Housing	8 (15%)	2 (4%)	3 (6%)	0 (0%)
Consumption	8 (15%)	2 (4%)	5 (9%)	0 (0%)
Legal or criminal justice	7 (13%)	3 (6%)	1 (2%)	0 (0%)
Scientific spillover	4 (8%)	0 (0%)	0 (0%)	2 (4%)
Reduction of uncertainty	4 (8%)	0 (0%)	1 (2%)	0 (0%)
Real option value	3 (6%)	0 (0%)	0 (0%)	1 (2%)
Economic activity	3 (6%)	0 (0%)	0 (0%)	2 (4%)
Value of hope	2 (4%)	0 (0%)	0 (0%)	1 (2%)
Healthcare capacity	2 (4%)	0 (0%)	0 (0%)	0 (0%)
Environment	1 (2%)	1 (2%)	0 (0%)	0 (0%)
Insurance value	0 (0%)	0 (0%)	0 (0%)	0 (0%)
Fear of contagion	0 (0%)	0 (0%)	0 (0%)	0 (0%)

a There were a total of fifty-three HTAs.

Abbreviation: HTA, health technology assessment.

### HTA guideline recommendations pertaining to societal and novel elements

Recommendations vary with regard to how HTAs should include the elements in their analysis. Some guidelines do not offer specific recommendations for measuring or including value elements and others provide varying levels of detail on the topic. Most guidelines do not recommend value element inclusion in the base case, sensitivity analysis, or qualitative discussion in the HTA. In our sample, the fifty-three HTA guidelines could have recommended the inclusion of each of the twenty-one value elements in the base case analysis for a total of 1,113 opportunities for an HTA guideline to recommend inclusion of a value element in the base case analysis. On average across all value elements included in our analysis, HTA guidelines recommend inclusion of a value element in the base case in 7 percent (seventy-seven) of those opportunities. Guidelines recommend inclusion of an element in the sensitivity analysis in 8 percent (eighty-eight) of those opportunities, and recommend qualitative discussion of an element in 4 percent (forty-six) of those opportunities. The elements that HTA guidelines most often recommended for inclusion in the base-case analysis are family spillover (34 percent), productivity (26 percent), and transportation (26 percent), followed by genericization (17 percent), social services (13 percent), and adherence-improving factors (9 percent). Fewer than 7 percent of HTA guidelines recommended the inclusion of the remaining elements in the base-case analysis. Patterns of recommendation vary by value element. For example, 43 percent of HTA guidelines (twenty-three of fifty-three) recommend including productivity in a sensitivity analysis, 26 percent (fourteen of fifty-three) recommend including productivity in the base case, and 4 percent (two of fifty-three) recommend including productivity in a qualitative discussion (Table 3). Thirty-four percent of HTA guidelines recommend including family spillover in the base case, and 34 percent recommend including this element in the sensitivity analysis (Table 3). Sixty-six percent of guidelines mention equity, although none of those guidelines recommends its inclusion in the base case analysis (Table 3). Only 9 percent of guidelines recommend including equity in the sensitivity analysis, whereas 43 percent recommend including equity in a qualitative discussion (Table 3).

### Inclusion of societal and novel value elements over time

**Figure 1.**
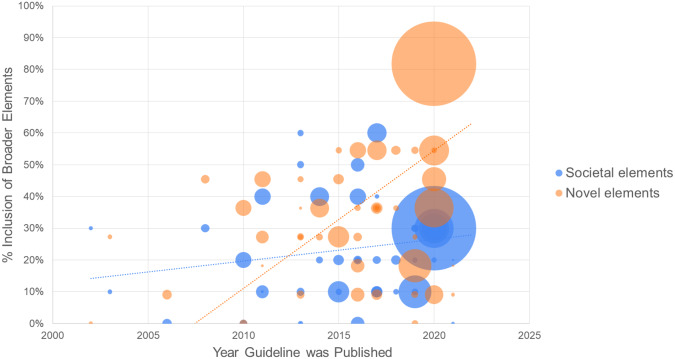
HTA guideline inclusion of societal and novel value elements over time. This figure reflects a linear regression weighted by country total health expenditure. The outcome is the proportion of guidelines including either societal or novel elements; the independent variable is the year the guideline was published. The proportion of societal and novel value elements included appears on average to be higher in guidelines published more recently.

The proportion of societal and novel value elements included appears on average to be higher in guidelines published more recently (Figure 1).

## Discussion

HTA organizations vary substantially in terms of the societal and novel elements of value they consider in their guidelines. Although mention of novel and societal elements appears to be growing over time, many guidelines still exclude them. When HTA guidelines do mention value elements, they infrequently recommend their inclusion in base case analysis, though some recommend their inclusion in sensitivity analysis and in qualitative discussions.

The quantitative inclusion of value elements in the calculation of a cost-effectiveness ratio rather than qualitatively alongside a ratio has advantages and potential downsides. Quantitative inclusion allows for explicit weighting of different value attributes, though it poses challenges due to the lack of consensus in the field (e.g., on how to include elements such as family spillover effects and equity) and a lack of data to support estimates. Including such elements qualitatively allows audiences to synthesize information in a more holistic manner, though it masks information about the weight assigned to different factors.

Gaps may exist between HTA organizations’ recommendations and actual practice in the jurisdictions they cover. As one example, ICER recommends inclusion of a modified societal perspective when “the societal costs of care for any disease are large relative to the direct healthcare costs, and… the impact of treatment on these costs is substantial…” (11). However, ICER’s 2020 assessment of remdesivir for COVID-19 excluded consideration of certain societal value elements, such as a COVID-19 treatment’s potential impact on people’s ability to return to work and on reducing COVID-19’s impact on the United States healthcare system’s capacity (66).

Debates about how expansive to make value assessments continue. Proponents of including societal and novel elements argue that conventional CEAs fail to account for important benefits conferred by healthcare interventions (67). Skeptics express concerns about augmenting traditional analyses, pointing to the potential for double counting elements, and a lack of suitable data. Some view the practice as undermining a more pragmatic healthcare system centric (i.e., “extra-welfarist”) approach for CEA (68;69).

Future research should investigate how HTA organizations handle the inclusion and measurement of societal and novel value elements (70) and how much inclusion of elements influences results. Shafrin et al. found that willingness to pay by healthy individuals for generous insurance coverage (insurance value) for new lung cancer treatments may represent almost 90 percent of the value for these treatments (71). In a study of the value of hope, Reed et al. found that, holding expected survival constant, participants would pay $6,446 to increase the chance of long-term survival from 5 to 10 percent (72). Multiple studies have estimated the real option value of a cancer drug that allowed patients to survive until the introduction of a new treatment, with estimates varying from .4 to 57 percent (73–76).

HTA organizations may omit societal and novel elements for a number of reasons. First, their purview may extend to payers with narrow remits to allocate fixed health budgets. Second, they may have significant concerns about the feasibility of estimating societal and novel elements. Less mature HTA organizations may lack the expertise and resources to assess societal and novel elements and would benefit from collaboration with larger HTA organizations. Possibly, efforts such as the EuNetHTA core model could help in these efforts.

Ideally, more HTA organizations will consider adopting guidelines for measuring societal and novel value elements, including analytic considerations (i.e., whether to include them in base case, in sensitivity analysis, or in qualitative discussion). To be sure, including non-healthcare value elements can be technically challenging and can add uncertainty and these factors must be considered. But such a step would more appropriately reflect the full consequences of using new technologies and is worth undertaking.

Importantly, simply recommending in guidelines that HTA bodies consider novel elements may not lead to their incorporation into assessments or ultimate decision making. Future research should explore to what extent HTAs include societal and novel value elements in practice, to what extent decision makers factor this information into their coverage and reimbursement decisions, and the implications for resource allocation. Finally, it will be important for manufacturers to design, develop, and communicate robust and pertinent evidence on societal and novel value elements related to their products across different dimensions, stakeholders, and sectors.

## Supplementary material

For supplementary material accompanying this paper visit https://doi.org/10.1017/S026646232300017X. click here to view supplementary material
