# Supplementary material for: A review of HTA guidelines on societal and novel value elements
Source: Int J Technol Assess Health Care. 2023 May 25;39(1):e31. doi: 10.1017/S026646232300017X (PMC11574534; doi:10.1017/S026646232300017X)
Supplement: Supplementary file 1 [file S026646232300017Xsup.zip › S026646232300017Xsup001.docx]

**Appendix Table 1.** Details of HTA Guidelines

| **Country** | **World Bank geographic region** | **World Bank Income Classification Group** | **HTA organization** | **Guideline title** | **Last updated** |
| --- | --- | --- | --- | --- | --- |
| Australia | East Asia & Pacific | High | Pharmaceutical Benefits Advisory Committee. Australian Government Department of Health | “Guidelines for preparing a submission to the Pharmaceutical Benefits Advisory Committee (Version 5.0)”  "Manual of resource items and their associated unit costs - December 2016" | 2016 |
| Austria | Europe & Central Asia | High | Institute for Pharmaeconomic Research (IPF) | “Guidelines on Health Economic Evaluation. Consensus paper” | 2006 |
| Baltic: Latvia, Lithuania, Estonia | Europe & Central Asia | High | Medicines’ Pricing and Reimbursement Agency, Latvia; Health Insurance Fund, Estonia; Department of Pharmacy under the Ministry of Health, Lithuania | “Baltic guideline for economic evaluation of pharmaceuticals” | 2002 |
| Belgium | Europe & Central Asia | High | Belgian Health Care Knowledge Centre (KCE) | “Belgian Guidelines for Economic Evaluations and Budget Impact Analyses: Second Edition” | 2012 |
| Brazil | Latin America & Caribbean | Upper middle | Brazil Ministry of Health | "Methodological Guidelines: Economic Evaluation of Health Technologies" | 2014 |
| Canada | North America | High | Canadian Agency for Drugs and Technologies in Health (CADTH) | “Guidelines for the Economic Evaluation of Health Technologies: Canada — 4th Edition” | 2017 |
| Chile | Latin America & Caribbean | High | Departamento de Economía de la Salud Subsecretaría de Salud Pública Ministerio de Salud de Chile | “Guía Metodológica para la Evaluación Económica de Intervenciones en Salud en Chile” | 2013 |
| China | East Asia & Pacific | Upper middle | Chinese Pharmaceutical Association, Chinese Society for Pharmacoeconomics and Outcomes Research, ISPOR Beijing Chapter | "China Guidelines for Pharmacoeconomic evaluations (2020 Edition)" | 2020 |
| Colombia | Latin America & Caribbean | Upper middle | Instituto de Evaluación Tecnológica en Salud – IETS | “Manual para la elaboración de evaluaciones económicas en salud” | 2014 |
| Croatia | Europe & Central Asia | High | Agency for Quality and Accreditation in Health Care, Croatia Department for Development, Research and Health Technology Assessment | “The Croatian Guideline for Health Technology Assessment Process and Reporting” | 2011 |
| Cuba | Latin America & Caribbean | Upper middle | Ministerio de Salud Pública. Escuela Nacional de Salud Pública | “Guía metodológica para la evaluación económica en salud. Cuba, 2003” | 2003 |
| Czech Republic | Europe & Central Asia | High | Státní Ústav pro Kontrolu Léčiv | "Procedure for assessing cost-effectiveness analysis" Original text: “Postup pro posuzování analýzy nákladové efektivity” | 2017 |
| Denmark | Europe & Central Asia | High | Danish Centre for Health Technology Assessment, National Board of Health | "Health Technology Assessment Handbook" | 2008 |
| Drummond et al. | N/A | N/A | N/A | "Methods for the Economic Evaluation of Health Care Programmes, Fourth Edition" | 2015 |
| Egypt | Middle East & North Africa | Lower middle | Ministry of Health and Population (MOHP) in Egypt. Pharmacoeconomic Unit | “Recommendations for Reporting Pharmacoeconomic Evaluations in Egypt” | 2013 |
| England & Wales | Europe & Central Asia | High | National Institute for Health and Care Excellence (NICE) | “Guide to the methods of technology appraisal 2013” | 2014 (updated 2022) |
| Finland | Europe & Central Asia | High | Pharmaceuticals Pricing Board, Ministry of Social Affairs and Health | Preparing a Health Economic Evaluation to Be Attached to the Application for Reimbursement Status and Wholesale Price for a Medicinal Product. Application Instructions (December 2019) | 2019 |
| France | Europe & Central Asia | High | Department of Economics and Public Health Assessment, Haute Autorité de Santé (HAS) | "Choices in Methods for Economic Evaluation" Original text: Choix méthodologiques pour l'évaluation économique à la HAS | 2020 |
| Germany | Europe & Central Asia | High | Institute for Quality and Efficiency in Health Care (IQWiG) | "General Methods" | 2020 |
| Hungary | Europe & Central Asia | High | National Institute of Pharmacy and Nutrition | “Professional Healthcare Guideline on the Methodology of Health Technology Assessment” | 2017 |
| Indonesia | East Asia & Pacific | Upper middle | Indonesian Health Technology Assessment Committee (InaHTAC), Ministry of Health, Indonesia | “Health Technology Assessment (HTA) Guideline.” | 2017 |
| International - ISPOR | N/A | N/A | International Society for Pharmacoeconomics and Outcomes Research | "Good Research Practices for Measuring Drug Costs in Cost Effectiveness Analyses: The ISPOR Drug Cost Task Force Report" Parts I-VI | 2010 |
| Ireland | Europe & Central Asia | High | Health Information and Quality Authority | “Guidelines for the Economic Evaluation of Health Technologies in Ireland” | 2019 |
| Israel | Middle East & North Africa | High | Ministry of Health Pharmaceutical Administration | “Guidelines for the submission of a request to include a pharmaceutical product in the national list of health services” | 2010 |
| Italy | Europe & Central Asia | High | Italian Group for Pharmacoeconomic Studies | LINEE GUIDA PER LA COMPILAZIONE DEL DOSSIER A SUPPORTO DELLA DOMANDA DI RIMBORSABILITÀ E PREZZO DI UN MEDICINALE | 2020 |
| Japan | East Asia & Pacific | High | National Institute of Public Health (C2H) | “Guideline for Preparing Cost-Effectiveness Evaluation to the Central Social Insurance Medical Council” | 2019 |
| Malaysia | East Asia & Pacific | Upper middle | Ministry of Health Malaysia, Pharmaceutical Services Programme | “Pharmacoeconomic Guidelines for Malaysia” | 2019 |
| MERCOSUR: Argentina, Brazil, Paraguay, Uruguay | Latin America & Caribbean | Upper middle/high (Uruguay) | Sistema Argentina de Información Jurídica, Grupo Mercado Común | "Guide for Economic Evaluation Studies of Health Technologies" Original text: “Guía Para Estudios de Evaluación Económica de Tecnologías Sanitarias” | 2015 |
| Mexico | Latin America & Caribbean | Upper middle | Comisión Interinstitucional del Cuadro Básico de Insumos del Sector Salud | "Guide for the Conduct of Economic Evaluation Studies for Updating the Basic Table and Catalog of Supplies of the Health Sector in Mexico." Original text: “Guía para la Conducción de Estudios de Evaluación Económica para la Actualización del Cuadro Básico y Catálogo de Insumos del Sector Salud en México.” | 2017 |
| Netherlands | Europe & Central Asia | High | National Health Care Institute | "Guideline for economic evaluations in healthcare" | 2016 |
| New Zealand | East Asia & Pacific | High | Pharmaceutical Management Agency (PHARMAC) | “Prescription for Pharmacoeconomic Analysis. Methods for cost-utility analysis” | 2015 |
| Norway | Europe & Central Asia | High | Norwegian Medicines Agency (Legemiddelverket) | “Guidelines for the Submission of Documentation for Single Technology Assessment (STA) of Pharmaceuticals” | 2018 |
| Philippines | East Asia & Pacific | Lower middle | Department of Health - Philippines | "Philippine Methods Guide for Health Technology Assessment (First Edition)" | 2020 |
| Poland | Europe & Central Asia | High | Agencja Oceny Technologii Medycznych i Taryfikacji | "Guidelines for health technology assessment (HTA)" Original text: “Wytyczne oceny technologii medycznych (HTA, ang. health technology assessment)” | 2016 |
| Portugal | Europe & Central Asia | High | Ministry of Health and the National Health Service | "Orientações Metodológicas para Estudos de Avaliação Económica de Tecnologias de Saúde" | 2019 |
| Russia | Europe & Central Asia | Upper middle | Federal State Budgetary Institution; Center for expertise and quality control of medical care, Ministry of Health of Russia | "Guidelines for conducting a comparative clinical and economic evaluation of drugs"; "Guidelines for assessing the budget impact n the framework of the Programm of the State Guarantee of Free Medical Care in the Russian Federation" | 2016 |
| Scotland | Europe & Central Asia | High | Scottish Medicines Consortium (SMC) | "Guidance to submitting companies for completion of New Product Assessment Form (NPAF)" | 2020 |
| Singapore | East Asia & Pacific | High | Ministry of Health. Agency for Care Effectiveness (ACE) | "Drug Evaluation Methods and Process Guide" | 2019 |
| Slovak Republic | Europe & Central Asia | High | Ministerstvo Zdravotníctva Slovenskej Republiky | "Methodical tool for performing pharmaco-economic analysis of a drug, medical-economic analysis of a medical device and medical-economic analysis of a dietetic food" Original text: “Metodická pomôcka pre vykonávanie farmako-ekonomického rozboru lieku, medicínsko-ekonomického rozboru zdravotníckej pomôcky a medicínsko-ekonomického rozboru dietetickej potraviny” | 2012 |
| Slovenia | Europe & Central Asia | High | Zavod za zdravstveno zavarovanje Slovenije | "Rules on the classification of medicinal products on the list" Original text: „Pravilnik o razvrščanju zdravil na listo” | 2013 |
| South Africa | Sub-Saharan Africa | Upper middle | Department of Health | "Guidelines for Pharmacoeconomic Submissions" | 2013 |
| South Korea | East Asia & Pacific | High | Health Insurance Review and Assessment Service (HIRA) | "Drug economic evaluation guidelines and data preparation method" Original text: 의약품경제성평가 지침 및 자료작성요령 | 2011 |
| Spain | Europe & Central Asia | High | Spanish Ministry of Health and Social Policy | “Spanish Recommendations on Economic Evaluation of Health Technologies” | 2010 |
| Sweden | Europe & Central Asia | High | The Swedish Dental and Pharmacetuical Benefits Agency; Tandvårds- och läkemedelsförmånsverket (TLV) | "Tandvårds- och läkemedelsförmånsverkets allmänna råd" | 2017 |
| Switzerland | Europe & Central Asia | High | Swiss Federal Office of Public Health | "Operationalisation of the Terms Effectiveness, Expediency and Profitability of Pharmaceuticals" | 2011 |
| Taiwan | East Asia & Pacific | Upper middle (China) | Taiwan Society for Pharmacoeconomics and Outcomes Research | “Guidelines of Methodological Standards for Pharmacoeconomic Evaluations in Taiwan” | 2006 |
| Thailand | East Asia & Pacific | Upper middle | Department of Health, Ministry of Public Health | “Guidelines for health technology assessment in Thailand (second edition): Recommendations for defining the scope of HTA” | 2017 |
| Tunisia | Middle East & North Africa | Lower middle | INEAS - National Authority for Assessment and Accreditation in Healthcare | "METHODOLOGICAL CHOICESFOR PHARMACO-ECONOMIC STUDIES AT INEAS" Original Text: CHOIX METHODOLOGIQUES POUR LES ETUDES PHARMACO-ECONOMIQUES A L’INEAS | 2021 |
| United States - AMCP | North America | High | AMCP | "The AMCP Format for Formulary Submissions (Version 4.0)" | 2016 |
| United States - ICER | North America | High | Institute for Clinical and Economic Review | "ICER’s Reference Case for Economic Evaluations: Principles and Rationale" | 2020 |
| United States - Second Panel | North America | High | Second Panel on Cost-Effectiveness in Health and Medicine | "Recommendations for Conduct, Methodological Practices, and Reporting of Cost-effectiveness Analyses" | 2016 |
| United States - WellPoint | North America | High | WellPoint | "The WellPoint Outcomes Based Formulary: enhancing the health technology assessment process" | 2005 |
| Wilkinson et al. | N/A | N/A | N/A | "The International Decision Support Initiative Reference Case for Economic Evaluation: An Aid to Thought" | 2016 |
