# Supplementary material for: A review of HTA guidelines on societal and novel value elements
Source: Int J Technol Assess Health Care. 2023 May 25;39(1):e31. doi: 10.1017/S026646232300017X (PMC11574534; doi:10.1017/S026646232300017X)
Supplement: Supplementary file 1 [file S026646232300017Xsup.zip › S026646232300017Xsup002.docx]

**Appendix Table 2. Data collection form**

**Table of contents**

1. Overview Information
2. Societal Value Elements
3. Novel Value Elements

**I. Overview information**

1. Name of HTA organization
   1. Guideline title
2. Countr(y/ies) represented
3. Year published

**II. Societal value elements**

1. Does the guideline mention or reference any societal value elements? Y/N
2. Consumption?
3. Social services?
4. Legal or criminal justice?
5. Education?
6. Housing?
7. Environment?
8. Family Spillover?
9. Transportation?
10. Economic activity (supply and demand side)?
11. Healthcare system capacity?
12. If yes to 1 a-i, where does the HTA guideline recommend that HTAs include the value element? Select all that apply.
    - - Base case
      - Sensitivity analysis
      - Qualitative discussion
      - Other

**III. Novel value elements**

1. **“Novel” value elements**
   1. Does the guideline mention or reference any “novel” value elements? Y/N
      1. Productivity
      2. Adherence improving factors
      3. Real option value?
      4. Scientific spillovers?
      5. Value of hope?
      6. Insurance value?
      7. Equity?
      8. Severity of disease?
      9. Fear of contagion & disease?
      10. Reduction of uncertainty?
      11. Does the guidelines mention or recommend including assumptions about future declines in drug prices given the genericization of drugs?
   2. If yes to 1 a-i, where does the HTA guideline recommend that HTAs include the value element? Select all that apply.
      - Base case
      - Sensitivity analysis
      - Qualitative discussion
      - Other
