# Supplementary material for: A review of HTA guidelines on societal and novel value elements
Source: Int J Technol Assess Health Care. 2023 May 25;39(1):e31. doi: 10.1017/S026646232300017X (PMC11574534; doi:10.1017/S026646232300017X)
Supplement: Supplementary file 1 [file S026646232300017Xsup.zip › S026646232300017Xsup003.docx]

Appendix 3. HTA guideline inclusion of individual societal and novel value elements

| Country | HTA Organization | Guideline title | Productivity | Adherence improving factors | Real option value | Scientific spillovers | Value of hope | Insurance value | Equity | Severity of disease | Fear of contagion & disease | Reduction of uncertainty | Consumption | Social services | Legal or criminal justice | Education | Housing | Environment | Family spillover | Transportation | Economic activity (supply and demand side) | Healthcare system capacity |
| --- | --- | --- | --- | --- | --- | --- | --- | --- | --- | --- | --- | --- | --- | --- | --- | --- | --- | --- | --- | --- | --- | --- |
| AMCP | Academy of Managed Care Pharmacy | The AMCP Format for Formulary Submissions Version 4.0 | Yes | Yes | No | No | No | No | Yes | Yes | No | No | No | No | No | No | No | No | Yes | No | No | No |
| Australia | Pharmaceutical Benefits Advisory Committee | Guidelines for preparing a submission to the Pharmaceutical Benefits Advisory Committee | Yes | Yes | No | No | No | No | Yes | Yes | No | Yes | No | No | Yes | Yes | Yes | No | Yes | No | No | No |
| Austria | Institute for Pharmaeconomic Research | Guidelines on Health Economic Evaluation: Consensus paper | Yes | No | No | No | No | No | No | No | No | No | No | No | No | No | No | No | No | No | No | No |
| Baltics (Latvia, Lithuania, Estonia) | Baltic Guideline for Economic Evaluation of Pharmaceuticals | Baltic Guideline for Economic Evaluation of Pharmaceuticals | No | No | No | No | No | No | No | No | No | No | No | Yes | No | No | No | No | Yes | Yes | No | No |
| Belgium | Belgian Health Care Knowledge Centre | BELGIAN GUIDELINES FOR ECONOMIC EVALUATIONS AND BUDGET IMPACT ANALYSES: SECOND EDITION | Yes | No | No | No | No | No | Yes | Yes | No | No | No | No | No | No | No | No | Yes | Yes | No | No |
| Brazil | Ministry of Health, Secretariat of Science and Strategic Inputs, Department of Science and Technology | Methodological Guidelines Economic Assessment Guideline | Yes | Yes | No | No | No | No | Yes | No | No | No | No | Yes | No | No | Yes | No | Yes | Yes | No | No |
| Canada | The Canadian Agency for Drugs and Technologies in Health | Guidelines for the Economic Evaluation of Health Technologies: Canada 4th Edition | Yes | Yes | No | No | No | No | Yes | Yes | No | Yes | No | Yes | Yes | Yes | Yes | No | Yes | Yes | No | No |
| Chile | Departamento de Economía de la Salud Subsecretaría de Salud Pública Ministerio de Salud de Chile (Department of Health Economics, Undersecretary of Public Health, Ministry of Health of Chile) | GUÍA METODOLÓGICA PARA LA EVALUACIÓN ECONÓMICA DE INTERVENCIONES EN SALUD EN CHILE (Methodologic guide for the economic evaluation of health interventions in Chile" | Yes | No | No | No | No | No | Yes | No | No | No | No | No | No | Yes | Yes | No | Yes | Yes | No | No |
| China | Chinese Pharmaceutical Association, China Society for Pharmacoeconomics and Outcomes Research, ISPOR Beijing Chapter Dec | China Guidelines for Pharmacoeconomic Evaluations | Yes | No | No | No | No | No | Yes | Yes | No | No | No | Yes | No | No | No | No | Yes | Yes | No | No |
| Colombia | Instituto de Evaluación Tecnológica en Salud – IETS | Manual for the elaboration of health economic evaluations (Manual para la elaboracion de evaluaciones economicas en salud) | Yes | No | No | No | No | No | Yes | No | No | No | No | No | No | No | No | No | Yes | Yes | No | No |
| Croatia | Agency for Quality and Accreditation in Health Care, Croatia Department for Development, Research and Health Technology Assessment | The Croatian Guideline for Health Technology Assessment Process and Reporting | No | No | No | No | No | No | Yes | No | No | No | No | No | No | No | No | No | Yes | No | No | No |
| Cuba | Ministry of Public Health, National School of Public Health, Economy Area | METHODOLOGICAL GUIDE FOR ECONOMIC EVALUATION IN HEALTH. CUBA. 2003 | Yes | No | No | No | No | No | Yes | No | No | No | No | No | No | No | No | No | Yes | No | No | No |
| Czech Republic | České farmakoekonomické společnosti (ČFES) | Recommended procedures of the Czech Pharmacoeconomic Society (ÿFES) for health and economic evaluations in the Czech Republic (Doporučené postupy České farmakoekonomické společnosti (ČFES) pro zdravotně-ekonomická hodnocení v ČR Pracovní skupina pro tvorbu doporučených postupů ČFES Duben) | Yes | No | No | No | No | No | Yes | Yes | No | No | No | No | No | No | No | No | Yes | Yes | No | No |
| Denmark | Danish Centre for Health Technology Assessment, National Board of Health | Health Technology Assessment Handbook | Yes | No | Yes | No | Yes | No | No | No | No | Yes | Yes | No | No | No | No | No | Yes | Yes | No | No |
| Drummond et al | Drummond et al. | Methods for the Economic Evaluation of Health Care Programmes, Fourth Edition | Yes | Yes | Yes | No | No | No | Yes | Yes | No | Yes | Yes | Yes | Yes | Yes | No | No | Yes | Yes | Yes | Yes |
| Egypt | Ministry of Health and Population, Egypt | Recommendations for Reporting Pharmacoeconomic Evaluations in Egypt | Yes | No | No | No | No | No | Yes | No | No | No | No | No | No | No | No | No | No | No | No | No |
| England | National Institute for Health and Care Excellence | Developing NICE guidelines: the manual | Yes | No | No | Yes | No | No | Yes | No | No | No | No | No | No | No | No | No | Yes | No | No | No |
| Finland | Pharmaceuticals Pricing Board (Lääkkeiden Hintalautakunta Läkemedelsprisnämnden) | PREPARING A HEALTH ECONOMIC EVALUATION TO BE ATTACHED TO THE APPLICA-TION FOR REIMBURSEMENT STATUS AND WHOLESALE PRICE FOR A MEDICINAL PRODUCT | Yes | No | No | No | No | No | No | No | No | No | No | Yes | No | No | No | No | Yes | Yes | No | No |
| France | French National Authority for Health (Haute Autorité de santé (HAS)) | Choices in Methods for Economic Evaluation to HAS (Choix méthodologiques pour l'évaluation économique à la HAS) | Yes | No | No | No | No | No | Yes | Yes | No | No | No | Yes | No | No | No | No | Yes | Yes | No | No |
| Germany | Institute for Quality and Efficiency in Health Care (IQWiG), Foundation for Quality and Efficiency in Health Care | General Methods | Yes | Yes | No | No | No | No | Yes | Yes | No | No | Yes | No | No | No | No | No | Yes | Yes | No | No |
| Hungary | The National Institute of Pharmacy and Nutrition (Országos Gyógyszerészeti és Élelmezés-egészségügyi Intézet (OGYÉI)) | PROFESSIONAL HEALTHCARE GUIDELINE ON THE METHODOLOGY OF HEALTH TECHNOLOGY ASSESSMENT | Yes | Yes | No | No | No | No | Yes | No | No | No | Yes | No | No | No | No | No | No | Yes | No | No |
| ICER | Institute for Clinical and Economic Review | 2020-2023 Value Assessment Framework | Yes | Yes | Yes | Yes | Yes | No | Yes | Yes | No | No | No | No | Yes | Yes | No | No | Yes | No | No | No |
| Indonesia | Indonesian Health Technology Assessment Committee (InaHTAC) Ministry of Health Republic of Indonesia | Health Technology Assessment (HTA) Guideline | Yes | Yes | No | No | No | No | Yes | No | No | No | No | No | No | No | No | No | Yes | Yes | No | No |
| Ireland | Health Information and Quality Authority | Guidelines for the Economic Evaluation of Health Technologies in Ireland | Yes | Yes | No | No | No | No | Yes | Yes | No | No | No | No | No | Yes | No | No | Yes | No | No | Yes |
| ISPOR | International Society for Pharmacoeconomic and Outcomes Research (ISPOR) | Good Research Practices for Measuring Drug Costs in Cost Effectiveness Analyses: Issues and Recommendations: The ISPOR Drug Cost Task Force Report—Part Ivhe_663 3.. | Yes | Yes | No | Yes | No | No | No | No | No | No | No | No | No | No | No | No | Yes | No | No | No |
| Israel | Ministry of Health, Pharmaceutical Administration | Guidelines for the submission of a request to include a pharmaceutical product in the national list of health services | No | No | No | No | No | No | No | No | No | No | No | No | No | No | No | No | No | No | No | No |
| Italy | Italian Pharmaceutical Agency | LINEE GUIDA PER LA COMPILAZIONE DEL DOSSIER A SUPPORTO DELLA DOMANDA DI RIMBORSABILITÀ E PREZZO DI UN MEDICINALE (GUIDELINES FOR THE COMPILATION OF THE DOSSIER IN SUPPORT OF THE APPLICATION REFUNDABILITY AND PRICE OF A MEDICINE) | Yes | No | No | No | No | No | No | No | No | No | No | Yes | No | No | No | No | Yes | Yes | No | No |
| Japan | Center for Outcomes Research and Economic Evaluation for Health, National Institute of Public Health | Guideline for Preparing Cost-Effectiveness Evaluation to the Central Social Insurance Medical Council | Yes | No | No | No | No | No | No | No | No | No | No | No | No | No | No | No | Yes | No | No | No |
| Malaysia | Ministry of Health, Malaysia | Pharmacoeconomic Guidelines for Malaysia | Yes | No | No | No | No | No | Yes | No | No | No | No | Yes | No | No | No | No | No | Yes | No | No |
| MERCOSUR (Argentina, Brazil, Paraguay, Uruguay) | Common Market Group | GUÍA PARA ESTUDIOS DE EVALUACIÓN ECONÓMICA DE TECNOLOGÍAS SANITARIAS (Guide for economic evaluation studies of health technology) | Yes | No | No | No | No | No | Yes | No | No | No | No | No | No | No | No | No | Yes | No | No | No |
| Mexcio | Comisión Interinstitucional del Cuadro Básico de Insumos del Sector Salud (Interinstitutional Commission of the Basic Table of Health Sector Inputs) | Guía para la Conducción de Estudios de Evaluación Económica para la Actualización del Cuadro Básico y Catálogo de Insumos del Sector Salud en México (Guide for the conduction of economic evaluation studies for the update of the basic table and catalog of supplies of the health sector in Mexico) | Yes | Yes | No | No | No | No | Yes | No | No | No | No | No | No | No | No | No | No | Yes | No | No |
| Netherlands | Zorginstituut Nederland (National Health Care Institute) | Guideline for economic evaluations in healthcare | Yes | Yes | No | No | No | No | No | No | No | No | No | Yes | Yes | Yes | No | No | Yes | Yes | No | No |
| New Zealand | Pharmaceutical Management Agency | Prescription for Pharmacoeconomic Analysis | Yes | Yes | No | No | No | No | Yes | Yes | No | No | No | No | No | No | No | No | Yes | No | No | No |
| Norway | Statens Legemiddelverk (The Norwegian Medicines Agency) | Guidelines for the submission of documentation for single technology assessment (STA) of pharmaceuticals | Yes | Yes | No | No | No | No | Yes | Yes | No | No | No | No | No | No | No | No | Yes | Yes | No | No |
| Philippines | Department of Health - Philippines | Philippine Methods Guide for Health Technology Assessment | Yes | Yes | No | No | No | No | Yes | Yes | No | No | No | No | No | No | No | No | Yes | Yes | No | No |
| Poland | The Agency for Health Technology Assessment and Tariff System | Health Technology Assessment Guidelines | Yes | No | No | No | No | No | Yes | No | No | No | No | No | No | No | Yes | No | Yes | Yes | No | No |
| Portugal | The National Health Technology Assessment System (SiNATS) | Principles and characterization of Methodological Guidelines for Studies of Economic Assessment of Health Technologies | No | No | No | No | No | No | No | No | No | No | No | No | No | No | No | No | Yes | No | No | No |
| Russia | State Budgetary Institution of the Ministry of Health of Russia | Methodological Recommendations for Carrying Out Comparative Clinico-Economic Evaluation | Yes | No | No | No | No | No | No | No | No | No | No | No | No | No | No | No | No | No | No | No |
| Scotland | Scottish Medicines Consortium | Guidance to submitting companies for completion of New Product Assessment Form (NPAF) | No | Yes | No | Yes | No | No | No | Yes | No | No | No | Yes | No | No | No | No | Yes | No | No | No |
| Second Panel | Second Panel on Cost-Effectiveness in Health and Medicine | Recommendations for Conduct,Methodological Practices, and Reporting of Cost-effectiveness Analyses Second Panel on Cost-Effectiveness in Health and Medicine | Yes | No | No | No | No | No | Yes | Yes | No | No | Yes | Yes | Yes | Yes | Yes | Yes | Yes | Yes | No | No |
| Singapore | Agency for Care Effectiveness | Drug evaluation methods and process guide | Yes | Yes | No | No | No | No | Yes | Yes | No | No | No | No | No | No | No | No | Yes | No | No | No |
| Slovak Republic | Ministry of Health of the Slovak Republic | Decree of the Ministry of Health of the Slovak Republic on the details of pharmaco-economic analysis of the drug | No | Yes | No | No | No | No | No | No | No | No | No | No | No | No | No | No | No | No | No | No |
| Slovenia | Institute of Public Health Insurance of Slovenia | Rules for Classification of Medicinal Products | No | Yes | No | No | No | No | Yes | Yes | No | No | No | No | No | No | No | No | No | No | No | No |
| South Africa | Department of Health, South Africa | MEDICINES AND RELATED SUBSTANCES ACT (101 of 1965) REGULATIONS RELATING TO A TRANSPARENT PRICING SYSTEM FOR MEDICINES AND SCHEDULED SUBSTANCES: PUBLICATION OF THE GUIDELINES FOR PHARMACOECONOMIC SUBMISSIONS | No | Yes | No | No | No | No | No | No | No | No | No | Yes | No | No | No | No | No | No | No | No |
| South Korea | Health Insurance Review & Assessment Service | Guidelines for economic evaluation of drugs and guidelines for writing | Yes | No | No | No | No | No | Yes | Yes | No | No | Yes | Yes | No | No | No | No | Yes | Yes | No | No |
| Spain | Spanish Ministry of Health and Social Policy | Spanish recommendations on economic evaluation of health technologies | Yes | Yes | No | No | No | No | Yes | No | No | No | No | Yes | No | No | No | No | Yes | No | No | No |
| Sweden | The Swedish Dental and Pharmaceutical Benefits Agency | Amendment to the Swedish Dental and Pharmaceutical Benefits Agency's general advice (TLVAR 2003: 2) on financial evaluations | Yes | No | No | No | No | No | No | No | No | No | No | No | No | No | No | No | Yes | No | No | No |
| Switzerland | Swiss Confederation Performance and Principles Committee (ELGK) | Operationalization of the terms effectiveness, expediency and economy | No | No | No | No | No | No | Yes | Yes | No | No | No | No | No | No | No | No | Yes | No | No | No |
| Taiwan | Taiwan Society for Pharmacoeconomics and Outcomes Research | Guidelines of Methodological Standards for Pharmacoeconomic Evaluations in Taiwan | Yes | Yes | No | No | No | No | Yes | No | No | No | Yes | No | No | No | Yes | No | Yes | Yes | Yes | No |
| Thailand | Subcommittee for Development of the National List of Essential Drugs | Health Technology Assessment Manual for Thailand, Issue 2, 2013 | Yes | Yes | No | No | No | No | Yes | Yes | No | No | Yes | No | Yes | No | Yes | No | Yes | Yes | Yes | No |
| Tunisia | National Agency for Health Assessment and Accreditation (INEAS) | Methodological Choices for Pharmaco-Economic Studies | Yes | Yes | No | No | No | No | No | No | No | No | No | No | No | No | No | No | Yes | Yes | No | No |
| WellPoint | WellPoint | The WellPoint Outcomes Based Formulary SM: enhancing the health technology assessment process | No | No | No | No | No | No | No | No | No | No | No | No | No | No | No | No | No | No | No | No |
| Wilkinson et al | Wilkinson et al | The International Decision Support Initiative Reference Case for Economic Evaluation: An Aid to Thought | No | No | No | No | No | No | Yes | No | No | No | No | No | No | No | No | No | No | No | No | No |
